# CD4+ T Cells Are Dispensable for Induction of Broad Heterologous HIV Neutralizing Antibodies in Rhesus Macaques

**DOI:** 10.3389/fimmu.2021.757811

**Published:** 2021-10-20

**Authors:** Sanghita Sarkar, David A. Spencer, Philip Barnette, Shilpi Pandey, William F. Sutton, Madhubanti Basu, Reuben E. Burch, John D. Cleveland, Alexander F. Rosenberg, Javier Rangel-Moreno, Michael C. Keefer, Ann J. Hessell, Nancy L. Haigwood, James J. Kobie

**Affiliations:** ^1^ Department of Medicine, Division of Infectious Diseases, University of Alabama at Birmingham, Birmingham, AL, United States; ^2^ Oregon National Primate Research Center, Oregon Health & Science University, Beaverton, OR, United States; ^3^ School of Public Health, University of Alabama at Birmingham, Birmingham, AL, United States; ^4^ Department of Microbiology, University of Alabama at Birmingham, Birmingham, AL, United States; ^5^ Department of Medicine, Division of Allergy, Immunology and Rheumatology, University of Rochester Medical Center, Rochester, NY, United States; ^6^ Department of Medicine, Division of Infectious Diseases, University of Rochester Medical Center, Rochester, NY, United States

**Keywords:** HIV - human immunodeficiency virus, vaccine, antibody, CD4+ T cell, B cell, rhesus, neutralizing

## Abstract

Induction of broadly neutralizing antibodies (bNAbs) is a major goal for HIV vaccine development. HIV envelope glycoprotein (Env)-specific bNAbs isolated from HIV-infected individuals exhibit substantial somatic hypermutation and correlate with T follicular helper (Tfh) responses. Using the VC10014 DNA-protein co-immunization vaccine platform consisting of gp160 plasmids and gp140 trimeric proteins derived from an HIV-1 infected subject that developed bNAbs, we determined the characteristics of the Env-specific humoral response in vaccinated rhesus macaques in the context of CD4+ T cell depletion. Unexpectedly, both CD4+ depleted and non-depleted animals developed comparable Tier 1 and 2 heterologous HIV-1 neutralizing plasma antibody titers. There was no deficit in protection from SHIV challenge, no diminution of titers of HIV Env-specific cross-clade binding antibodies, antibody dependent cellular phagocytosis, or antibody-dependent complement deposition in the CD4+ depleted animals. These collective results suggest that in the presence of diminished CD4+ T cell help, HIV neutralizing antibodies were still generated, which may have implications for developing effective HIV vaccine strategies.

## Introduction

Thirty-eight million people are currently living with HIV, a prevalent virus that has claimed more than 34 million lives as of 2020. HIV infection remains a major global public health issue (1). Although 27.4 million people living with HIV infection received anti-retroviral therapy in 2020, the continued incidence of new HIV infections clearly shows the critical need for an effective HIV vaccine. A primary goal of HIV vaccine development is to induce sustained broadly neutralizing antibodies (bNAbs) capable of recognizing the substantial antigenic diversity of the HIV Envelope (Env) glycoprotein. Isolation and characterization of bNAbs from HIV infected patients have provided valuable insight into the various conserved B cell epitopes on HIV-1 Env (2, 3). Indeed, the molecular similarities between many of these antibodies and their evolution during the natural HIV-1 infection process has led to the design of immunogens to recapitulate the induction of broadly neutralizing antibodies that occurred in the HIV-1 patients (4, 5).

The binding of HIV gp120, *via* its highly conserved CD4 binding site (CD4bs), to CD4 on the host cell surface induces major conformational changes in Env, facilitating the binding of gp120 to either co-receptor CXCR4 or CCR5 and subsequent entry (6). The HIV-CD4+ T cell relationship is multi-faceted. Even though HIV-specific CD4^+^ T cells assist both HIV-specific CD8^+^ T cell and B cell responses by promoting strong cytotoxic T lymphocyte (CTL) activity and production of HIV-specific Abs (7), activated CD4^+^ T cells are targets for HIV infection and replication at mucosal sites (8). This complexity is a challenge to HIV-vaccine development. Based on efficacy trials that have utilized viral vector-based immunogens that typically induce cell mediated immunity such as HVTN 502/STEP (9), HVTN 503/Phambili (10) and HVTN 505 (11), it remains unclear to what extent excessive CD4+ T cell activation may contribute to the increased risk of infection or explain the lack of protection observed in these studies (12). A physiologic balance may be necessary to develop protective humoral immunity to HIV-1. Thus, priming strategies focused solely on driving high magnitude B cells responses coupled with high magnitude helper T cell responses may negate any humoral benefit by increasing viral targets. Accordingly, strategies that promote the induction of robust humoral immunity without excessive HIV-specific CD4+ T cell memory development may result in enhanced efficacy.

Several observations suggest that CD4+ T cell help may hinder the development of broad Ab responses. Correlative analysis of HIV bNAb occurrence in HIV infected patients by several groups showed increased serum HIV bNAb activity strongly correlated with decreased CD4+ T cells rather than increased viral load (13, 14). Consistent with this interpretation, HIV bNAbs occasionally develop within one year of infection, and at least as commonly in HIV-infected infants as adults (15) despite the well described deficits in CD4+ T cell function that are present during early life (16). In mice, CD4+ T cell depletion enhanced CD8+ T cell breadth and function in response to infection with murine gamma herpesvirus (17). Similarly, lack of T follicular helper (Tfh) cells and reduction in germinal center B cells, due to genetic deletion of BCL6, increased the magnitude and avidity of mouse gp120 Abs in response to HIV gp120 DNA/protein immunization (18), suggesting that an excessive CD4+ T cell response may undermine the quality of the B cell response to HIV envelope.

In this study, we utilized the VC10014 DNA-protein co-immunization vaccine platform consisting of gp160 plasmids and trimeric gp140 proteins derived from a clade B HIV-1 infected subject who developed broadly neutralizing plasma Abs, which previously induced Tier 2 heterologous neutralizing Abs in rabbits and rhesus macaques (19–21). We evaluated the influence of CD4+ T cell depletion during the vaccination regimen on the characteristics of the Env-specific humoral response in rhesus macaques. Both CD4+ T cell-depleted and non-depleted animals developed comparable Tier 2 heterologous neutralizing plasma antibodies. Similarly, there was no inferiority in titers of HIV Env-specific cross-clade binding antibodies, antibody-dependent cellular phagocytosis, or antibody-dependent complement deposition in the CD4+ depleted animals. These results suggest that in the absence of robust CD4+ T cell help, primates still generate HIV neutralizing antibodies, which may have implications for developing effective HIV vaccine strategies.

## Results

### Study Design and CD4 Depletion in Rhesus Macaques

**Figure 1:**
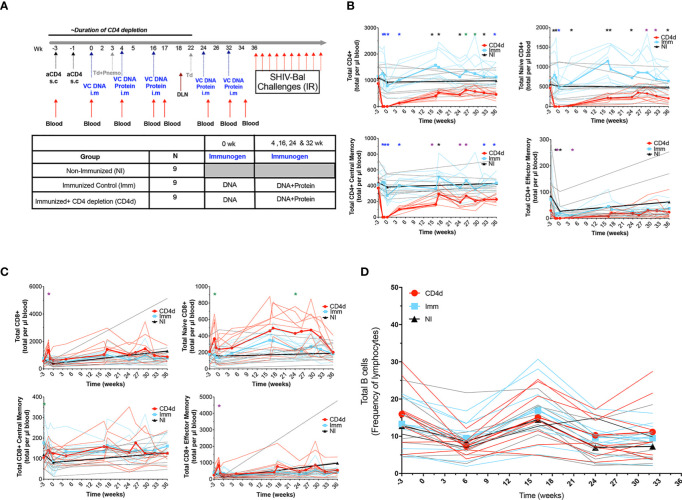
Frequencies of immune cell subsets in CD4 depleted and vaccinated rhesus macaques. **(A)** Immunization schedule: 27 adults rhesus macaques were assigned to this study and divided into two groups of nine for immunizations and the third group with nine macaques without immunization. All 18 rhesus macaques received VC10014 DNA plasmids and were then boosted i.m with co-immunization of VC10014 DNA plasmids and proteins in adjuplex. Nine rhesus macaques received CD4R1 during the priming phase. Flow cytometric analysis of longitudinal distribution of CD4+ **(B)** and CD8+ **(C)** T cells and total CD19+CD20+ B cells **(D)**. Red=CD4 depleted (CD4d) group, turquoise =Immunized (Imm) group, black=Non-Immunized (NI) group. Thin lines represent individual animals, thick lines indicated group mean. Significance determined by the unpaired t-test comparing Imm and CD4d groups and are indicated as follows: ^
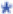
^p ≤ 0.001, *p ≤ 0.005, ^
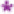
^p ≤ 0.01, ^
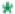
^p ≤ 0.05.

Using the Clade B VC10014 HIV-1 Env-based immunogens, rhesus macaques were primed with VC10014 gp160 plasmid DNA at week 0 and boosted with gp160 plasmid DNA and gp140 HIV envelope proteins at week 4, 16, 24 and 32 (**Figure 1A**). Nine adult rhesus macaques were subcutaneously injected at week -3 and week -1 before the first immunization with the CD4R1 mAb for transient depletion of CD4+ T cells (CD4d). The second group of nine adult rhesus macaques did not receive CD4R1 mAb but were immunized (Imm). An additional nine rhesus macaques were neither treated with CD4R1 mAb nor immunized (NI). The CD4d and Imm animals also received tetanus toxoid and Pneumovax to monitor prototypical CD4-dependent and CD4-independent vaccine responses. All rhesus macaques were repeatedly challenged with low dose SHIV-BaL intrarectally (IR) following the last vaccination, starting at week 36. Flow cytometry was done to evaluate CD4+ T cells depletion and reconstitution in the peripheral blood and whether CD4+ T cell depletion has an effect on the frequency of CD8+ T cells and B cells in the peripheral blood. The CD4d group experienced an 84% reduction in total CD4+ T cells through week 4 corresponding to the first two immunizations, and a 49% reduction in total CD4+ T cells through week 16 corresponding to the first three immunizations. The total CD4+ T cells slowly increased to ~70% of baseline levels at week 32, the time of the final immunization (**Figure 1B** and **Supplemental Figure 1**). We found a significant depletion of all CD4+ T cell subsets, including naïve, central memory, and effector memory. There was no significant change in total CD8+ T cells, naïve CD8+ T cells, CD8+ effector memory or central memory CD8+ T cells among the groups (**Figure 1C**). There was no statistically significant change in B cells amongst the groups (**Figure 1D**). Collectively, these data suggest that the CD4R1 mAb was effective in depleting CD4+ T cells during the early stages of immunization without affecting other CD8+ T cells or B cells.

### CD4 Depletion Inhibits the Germinal Center Response

**Figure 2:**
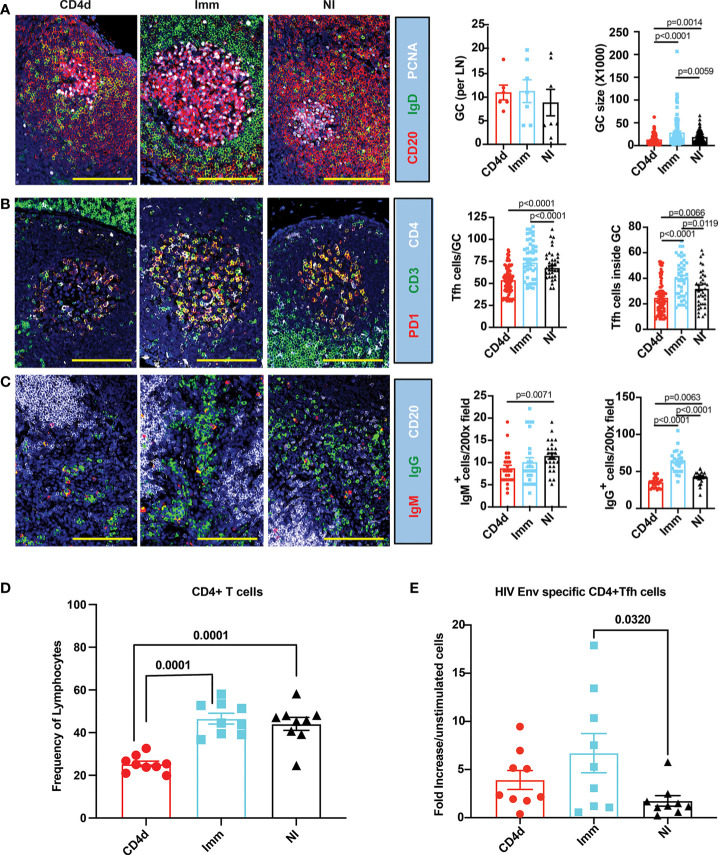
CD4 depletion impairs the germinal center responses in HIV-vaccinated rhesus macaques. Inguinal lymph nodes from CD4d, Imm and NI rhesus macaques were biopsied at week 18 following immunization to visualize and measure GCs, and enumerate CD3+CD4+PD1+ T follicular helper cells (Tfh) and IgG+/IgM+ plasma cells in 200X random fields by immunofluorescence staining. **(A)** Representative images of lymph nodes looking at proliferating GCs labeled with PCNA (white), IgD (green), and CD20 (red). **(B)** Representative images of CD3+CD4+PD1+ Tfh inside and outside the GCs of lymph nodes. CD4 (white), CD3 (green) and PD1(red). **(C)** Representative images of IgM^+^ and IgG^+^ plasma cells in lymph nodes identified by CD20 (white), IgG (green), and IgM (red). Representative pictures at 200x magnification. Scale bar represents 100 mm. **(D)** CD4+ T cells were measured by flow cytometry at week 18 in the inguinal lymph nodes. **(E)** HIV Env-specific CD4+ Tfh cell response at week 34 in peripheral blood in the CD4d group at weeks 34, 2 weeks after the last immunization was determined by AIM assay and indicates fold increase in the frequency in gp140 stimulated cultures over that in unstimulated cultures. Significance was calculated with two-tailed unpaired Student’s t-test. p < 0.05 was considered significant.

To assess the impact of CD4 depletion on the induction of the germinal center (GC) response, draining lymph nodes were collected at week 18 (2 weeks following the third immunization) and immunohistochemistry analysis was performed. There was a trend toward an increased number of GC in both immunized groups compared to NI. However, GCs were significantly larger (p=0.0059) in the Imm group compared to NI, and markedly smaller in the CD4d group compared to NI (p=0.0014) and Imm (p<0.0001) groups (**Figure 2A**). CD3+CD4+PD1+ T follicular helper cells (Tfh) associated with GC were significantly increased in Imm (p<0.0001), particularly inside the GC compared to NI (**Figure 2B**). As expected, the CD4d group had significantly less Tfh inside the GC compared to NI (p=0.0066) and Imm (p<0.0001) groups. IgM+ B cells were significantly decreased in the CD4d group compared to NI (p=0.0071) (**Figure 2C**). IgG+ B cells were significantly increased (p<0.0001) in the Imm group compared to NI, while they were less numerous in the CD4d group (p=0.0063). These results indicate that CD4 depletion substantially compromises the CD4+ Tfh and GC B cell responses to the vaccination. Consistent with the immunohistochemistry analysis, flow cytometry of the draining lymph nodes confirmed a ~50% reduction in CD4+ T cells in the CD4d group that was significant (p<0.0001) compared to the Imm and NI groups (**Figure 2D**). Consistent with an impaired GC response at week 18, a diminished peripheral blood HIV Env-specific CD4+ Tfh cell response in the CD4d group was still evident at week 34 (2 weeks after the last immunization), which was not significantly greater than the NI group in contrast to the Imm group (**Figure 2E**). Thus, CD4 depletion, although probably incomplete, impaired the GC response and the induction of HIV Env-specific CD4+ T cells, a deficit that persisted throughout the rest of the study.

### CD4 Depletion Does Not Affect the Development of Plasma HIV Env, Tetanus, and Pneumococcal Antibodies

**Figure 3:**
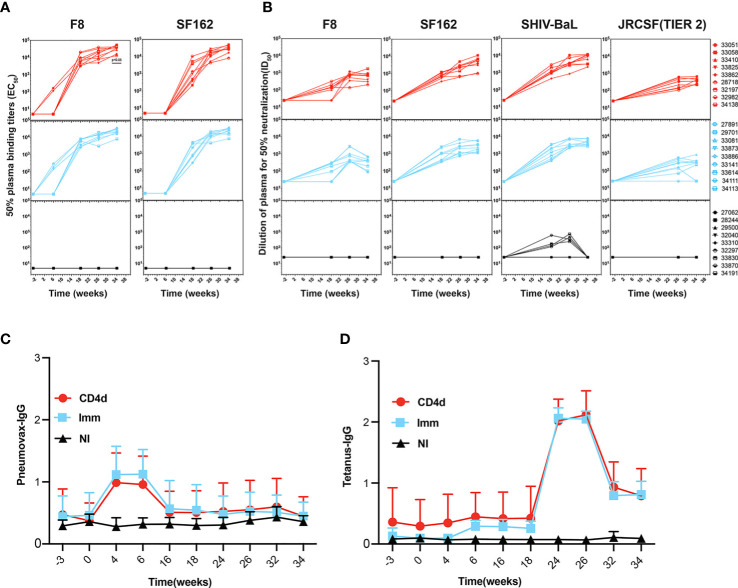
Rhesus macaques develop longitudinal autologous and heterologous Env-specific antibodies. **(A)** F8-gp140 and SF162-gp140 specific plasma IgG was determined by ELISA. Symbol represented individual rhesus macaques. **(B)** Plasma samples from VC10014-vaccinated macaques were tested longitudinally for neutralization antibodies to F8, SF162, SHIV-BaL, and JRCSF (Tier 2) by TZM-bl assay. Longitudinal ELISA showing Pneumovax **(C)** and Tetanus **(D)** specific plasma IgG. Red=CD4 depleted (CD4d) group, turquoise =Immunized (Imm) group, black=Non-Immunized (NI) group. Significance is determined by two-tailed unpaired t-test (p ≤ 0.05).

Plasma IgG binding antibodies to autologous F8 gp140 and heterologous SF162 gp140 throughout the course of immunization were measured by ELISA. As early as week 6, which is just after the first VC10014 DNA and protein immunization, F8-specific IgG was evident in both CD4d and Imm groups and by week 18 (2 weeks after the second VC10014 DNA and protein immunization) heterologous SF162 specific IgG was present both in CD4d and Imm groups. CD4+ T cell depletion did not affect F8 or SF162 IgG titers (**Figure 3A**). We measured neutralizing antibodies (NAbs) in the plasma of vaccinated macaques against a panel of both autologous (F8) and heterologous clade B Tier 1A (SF162) and clade B, Tier 1B (BaL) pseudoviruses, including the clade B Tier 2 virus, JRCSF (**Figure 3B**). NAbs against SF162 and SHIV-BaL were detected in all immunized animals at week 18, with F8 NAbs evident in 7/9 CD4d and 6/9 Imm animals at week 18. No inferiority in NAb titers against F8, SF162, or SHIV-BaL was evident in CD4d group at week 34, and notably, all CD4d animals had detectable Tier 2 JRCSF NAbs at this timepoint compared to their occurrence in only 7/9 Imm animals. There were no significant differences in Pneumovax (**Figure 3C**) or tetanus toxoid IgG titers (**Figure 3D**) between the CD4d and Imm groups. These results indicate that VC10014 can induce heterologous Tier 2 NAbs, and CD4 depletion does not hinder the development of HIV Env-specific binding or neutralizing plasma Abs.

### Induction of Broad and Poly-Functional Antibodies

**Figure 4:**
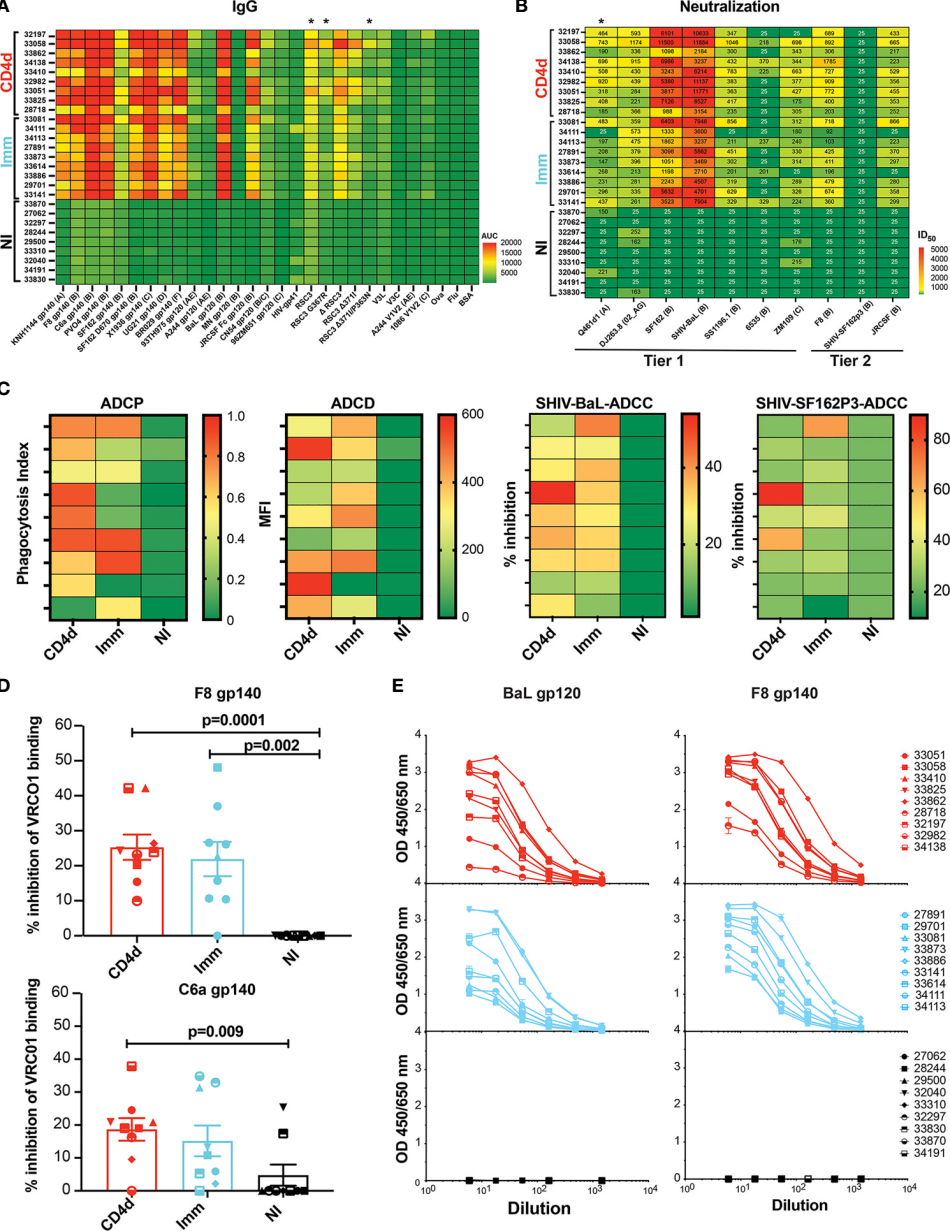
Broad antibody response at week 34. **(A)** Overview of broad HIV-Envelope specific IgG response at week 34. **(B)** Heterologous neutralizing antibodies elicited by VC10014 DNA plasmid and protein vaccine regimen. Rhesus plasma samples at week 34 were tested for neutralization in a TZM-bl assay. Neutralization data are expressed as ID_50_, the plasma dilution that neutralized 50% of the infecting virus. **(C)** Heat maps showing Ab-dependent cellular phagocytosis (ADCP, left panel), Ab-dependent complement deposition (ADCD, middle panel) and Ab-dependent cellular cytotoxicity (ADCC, right two panels) between CD4d, Imm, and NI groups. **(D)** Competition ELISA showing the inhibition of CD4bs-specific VRC01 mAb to HIV envelope in rhesus plasma. **(E)** ELISA showing autologous F8 gp140 specific IgG and heterologous Bal gp120 specific IgG in buccal mucosa of CD4d, Imm, and NI rhesus macaques. Red = CD4 depleted (CD4d) group, turquoise = Immunized (Imm) group, black = Non-Immunized (NI) group. * indicates significance (p < 0.05) as determined by unpaired t-test comparing Imm and CD4d groups.

To evaluate in-depth the breadth and diversity of the plasma antibody resulting from the VC10014 regimen, the binding Ab response was profiled at week 34 (2 weeks after the last immunization). There was no significant difference in the gp140, gp120, or gp41 IgG specific titers between the CD4d and Imm groups (**Figure 4A**). There was no appreciable change in HIV-Env specific IgM and IgA responses amongst the CD4d and Imm groups (**Supplemental Figures 2A, B**). The CD4d and Imm animals developed broad cross-clade Tier 1 neutralizing Ab titers, with no significant diminishment of neutralizing Ab titers in the CD4d group (**Figure 4B**). The CD4d group had significantly higher titers to Tier 1 clade A Q461d1 (p=0.02) than the Imm group. Both groups developed Tier 2 neutralizing antibodies including against autologous F8 and heterologous and JRSCF clade B viruses, with no reduction in the CD4d group. Notably, all animals in the CD4d group developed appreciable titers to JRCSF compared to only 7/9 animals in the Imm group. We next determined the ability of the plasma Abs to mediate Fc-effector functions. There was no significant difference in the antibody-dependent cellular phagocytosis (ADCP), antibody-dependent complement deposition (ADCD), or antibody-dependent cellular cytotoxicity (ADCC) activities between the CD4d and immunized groups (**Figure 4C**).

As a probe for detecting CD4bs-specific Abs in macaque IgG, we used the resurfaced stabilized gp120 core (RSC3), and CD4bs knockout variants, RSC3 G367R (22, 23) and RSC3 Δ371I/P363N (24, 25) to define the CD4bs plasma Ab response. Higher reactivity to the RSC3 and RSC3 G367R proteins can distinguish between CD4bs-specific bNAbs and other CD4bs mAbs that display weak or no binding (25). The CD4d group had a significantly greater response to all three of the RSC3 probes compared to the Imm group (RSC3, p = 0.0097; RSC3 G367R, p = 0.0290; and RSC3 Δ371I/P363N, p=0.03) (**Figure 4A**), suggesting enhanced targeting of the CD4bs in the CD4 depleted macaques. To further assess the presence of CD4 binding site Abs, plasma was tested for inhibition of the CD4bs-specific VRC01 bNmAb binding to Env. A trend toward greater inhibition of VRC01 binding was observed with plasma from the CD4d group compared to that in the Imm group (**Figure 4D**). Both CD4d and Imm animals developed buccal mucosal autologous (F8 gp140) and heterologous (BaL gp120) IgG antibodies, with no inferiority in IgG titers with CD4 depletion (**Figure 4E**). Together these results suggest that CD4 depletion does not impair the development of HIV Env-specific heterologous systemic and mucosal Abs, and CD4d may enhance the CD4bs-specific Ab response.

### CD4 Depletion During Immunization Reduces Viral Burden

**Figure 5:**
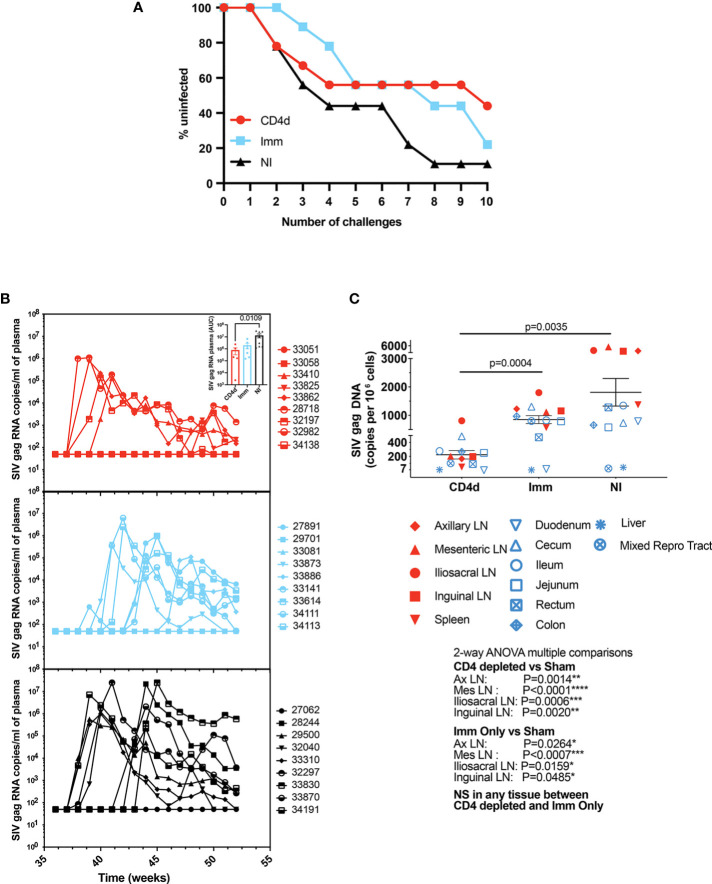
CD4 depletion during priming phase reduces risk of infection. **(A)** Rhesus macaques were challenged intrarectally with increasing doses of SHIV-BaL virus. Survival curves showing CD4 depletion reduces risk of infection. **(B)** RT-PCR was used to quantitate viral SHIV-BaL RNA in plasma. **(C)** Assessment of tissue-related virus (copies per million cells). Red = CD4 depleted (CD4d) group, turquoise = Immunized (Imm) group, black = Non-Immunized (NI) group. Significant difference (p < 0.05) between groups as determined by two-tailed t-test and 2-way ANOVA, *p < 0.05, **p <0.005, ***p < 0.001, ****p < 0.0001.

Next, we wanted to determine whether CD4 depletion during the early stages of the vaccination regimen alters protection from infection. Rhesus macaques were repeatedly challenged intrarectally with increasing doses of heterologous SHIV-BaL starting at week 36 (4 weeks after last immunization). After eight challenges, 1/9 NI, 3/9 Imm, and 5/9 CD4d animals remained un-infected (**Figure 5A**). After ten challenges, 1/9 NI, 2/9 Imm and 4/9 CD4d animals were protected. However, there was no significant difference in acquisition between groups. Among the animals that became infected, there was a trend toward decreased plasma viral burden in the immunized animals that reached significance for the CD4d group (p=0.0109) (**Figure 5B**). There was significantly lower overall viral burden in the tissues of the CD4d group as compared to the Imm group (p=0.0004) and NI group (p=0.0035) (**Figure 5C**). Among individual tissues there was significantly reduced viral burden in both the CD4d and Imm groups compared to the NI group in the axillary, mesenteric, iliosacral, and inguinal lymph nodes. These results suggest that the initial depletion of CD4+ T cells combined with immunization reduces risk of infection and subsequent viral burden following infection.

## Discussion

Defining the requirements for inducing protective Ab responses is essential for HIV vaccine development. The current dogma proposes that the induction of protective humoral immunity relies on robust CD4+ T cell helper responses. However, our results suggest CD4+ T cell help is dispensable for inducing robust B cell and antibody responses to HIV Env. We demonstrated that treatment with CD4 depleting Ab prior to immunization resulted in substantial depletion of CD4+ T cells in the periphery and lymph nodes, impairing the GC and Tfh cell response and induction of HIV Env-specific CD4+ T cell memory. Abrogating CD4+ T cell help did not impair antibody titers, breadth, or function, including the development of Tier 2 heterologous NAbs, and enhanced the development of CD4bs-specific Abs. These results highlight a precarious dichotomy of CD4+ T cells and development of humoral immunity to HIV.

Tfh participate in the development of memory B cells, long-lived plasma cells, and the selection of B cell clones that produce high affinity antibodies (26–28). The lack of any evident impairment in the humoral response despite a profound deficit in the Tfh and CD4+ helper response suggests either that only a minimal threshold of CD4+ help is required to achieve the optimal humoral response, or compensatory mechanisms may overcome a weak CD4+ T cell response. Highly repetitive molecules like the polysaccharide in the pneumococcal vaccine trigger T-independent B cell and antibody responses (29–31). Consistent with this process, a robust IgG response against pneumococcal vaccine was generated in both the CD4d and Imm groups. In contrast, conjugate vaccines such as tetanus toxoid depend on the help of T cells (32, 33). Surprisingly, no impairment of IgG response against tetanus toxoid was evident in the CD4d group. The B1 B cell response has minimal dependence on CD4+ T cell help, and although dominated by IgM in the early stages can result in class-switched IgG and IgA B cell and Ab development as a result of subsequent antigen exposure (34–36). Although the end result of this process, in this case development of HIV Env-specific IgG binding, Fc-effector function mediating and neutralizing Abs may grossly appear the same in the CD4d group, it is possible these developed as a result of a different initial B cell response (e.g. B1 *vs* B2). However, this study did not track the HIV Env-specific B cell response in sufficient resolution to discriminate this possibility, and it should be examined in future studies including if IgM and IgA antibodies contribute to neutralization.

The induction of CD4bs targeting neutralizing Abs is likely to be essential to developing an HIV vaccine that confers sufficient breadth to achieve efficacy. The mechanism by which CD4 depletion enhanced the development of CD4bs Abs is unclear and warrants further examination including if it impacts the breadth of Tier 2 neutralization. It can be speculated that CD4+ T cells may bind HIV Env immunogen, reducing its availability and partially occluding the CD4 binding site, limiting access for BCR and Ab recognition by some CD4bs specific B cells and Abs. The enhanced development of CD4bs specific Abs by CD4 depletion may also be a consequence of a decrease in the targeting of immunodominant epitopes driven in part by vigorous CD4+ T cell help, and a resulting increase in the diversity of the resulting Env-specific BCR repertoire and epitopes recognized. It is important to consider that the development of CD4bs bNAbs in HIV-1 infected people typically occurs in the context of compromised CD4+ T cell numbers and function (37, 38). Subsequently, these results clearly indicate that further resolution of the influence of CD4 binding to HIV Env immunogens and the consequences of CD4+ T cell help on the development of CD4bs is necessary as this dynamic may be a determinant of inducing broadly neutralizing CD4bs specific Abs by vaccination.

The VC10014 immunogen platform has consistently demonstrated the induction of Tier 2 heterologous NAbs (20, 21, 39). Although modest, the Imm group showed a trend toward enhanced protection from repeated heterologous challenge, as well as conferring decreased viral burden upon infection. Further optimizing the VC10014 immunogen platform, such as using Env modified for more native-like conformation, switching from DNA plasmid to MVA or mRNA delivery, or using a different adjuvant may improve the immunogenicity and ultimate efficacy. Further, as CD4 depletion is not a viable clinical strategy for HIV vaccine development, rational modification to de-optimize CD4+ T cell helper epitopes in the Env immunogens or structural modifications to eliminate CD4+ T cell binding such as previously reported (40) may be avenues for translation of our findings to advancing rational HIV vaccine development. Subsequently we are pursuing such efforts.

After ten challenges, four of the CD4d group remained uninfected, and those that did become infected had significantly lower viral burden than the NI and Imm groups. The lack of a significant difference in acquisition of infection between CD4d group and Imm group is likely a result of insufficient number of animals per group for that degree of resolution. Although challenges began more than nine months after administration of the CD4 depleting antibody, CD4+ T cell levels had not returned completely to baseline levels, which may have at least partly contributed to the increased protection observed. Similarly, the sustained impact of CD4 depleting Ab on the mucosal CD4+ T cell and mucosal CD4+ macrophage population is unclear and was not resolved in this study. Future experiments that include CD4 depletion only in the absence of immunization, and with mucosal sampling prior to challenge would help dissect this multifactorial relationship. This study does highlight the necessity to precisely define the contribution of and possible threshold levels of vaccine induced HIV Env-specific CD4+ T cells and global CD4+ T cells that may increase risk of HIV infection.

In summary, our results indicate that robust HIV Env-specific humoral response, including the development of Tier 2 heterologous neutralizing Abs can be generated in the presence of a compromised CD4+ T cell response. Our results further suggest that limiting CD4+ T cell help may qualitatively alter the HIV Env-specific B cell response including enhancing the development of CD4bs Abs. Thus, the development of an effective HIV vaccine will likely be dependent on finer resolution and manipulation of the CD4+ T cell and HIV-Env specific B cell dynamics.

## Materials and Methods

### Animals

The study was carried out in accordance with the recommendations described in the Guide for the Care and Use of Laboratory Animals of the National Institutes of Health and the United States Department of Agriculture. All animal work was approved by the Oregon Health & Science University (OHSU) West Campus Institutional Animal Care and Use Committee. Animal facilities at the Oregon National Primate Research Center (ONPRC) are accredited by the American Association for Accreditation of Laboratory Animal Care. All efforts were made to minimize animal suffering and all procedures involving potential pain were performed with the appropriate anesthetic or analgesic. The number of animals used in this study was scientifically justified based on statistical analyses of virological and immunological outcomes. Adult male and female Indian-origin *Macaca mulatta* (rhesus macaques) between 3.3 and 8.0 years of age were housed at the Oregon National Primate Research Center (Beaverton, OR) (**Supplementary Table 1**). All macaques were negative for MHC class I alleles Mamu B*08 and B*17. All procedures were performed according to regulations and protocols approved by the OHSU West Campus Institutional Animal Care and Use Committee.

### Immunization and Challenge

Nine rhesus macaques were CD4 depleted with 50 mg/kg and 25mg/kg of CD4R1 (NHP Reagent Resource Cat # PR-0407, RRID:AB_2716322) subcutaneously (s.c) on week -3 and -1, respectively. Eighteen rhesus macaques were immunized with HIV-1 VC10014 env gene expression plasmids at weeks 0, 4, 16, 24 and 32. At each immunization, a total of 36 µg of gp160 plasmid DNA was given intradermally (I.D) with a particle-mediated epidermal delivery (PMED) device (Gene Gun, XR-1 research model; PowderMed, Oxford, (UK). At weeks 4, 16, 24, and 32 animals were co-immunized with 100 µg recombinant Env gp140 protein total (50 μg each of F8 and C6a), formulated with 20% Adjuplex (Sigma) adjuvant, and delivered intramuscularly (I.M). The VC10014 immunogens have been previously described (5, 20). Briefly, the gp140 DNA for gp140 protein production was derived from the gp160 env sequence by site-directed mutagenesis (Stratagene, La Jolla, CA) to insert the previously described mutations (5, 41) in the primary and secondary protease cleavage sites respectively: REKR → RSKS and KAKRR → KAISS. A large-scale endotoxin-free plasmid preparation (Qiagen, Valencia, CA) was used for stable expression in 293F cells for protein production as described previously (42). Epitope exposure and antigenicity of gp140 trimeric protein immunogens was assessed by ELISA, biolayer interferometry (BLI), and surface plasmon resonance for binding of multiple bNmAbs as described previously (19). All 18 adult rhesus macaques also received Pneumovax 23 (Merck) and Tetanus-Diphtheria Toxoids Adsorbed (Td) (MassBiologics) I.M at week 3 and a Td boost at week 22. Peripheral blood was collected into Vaccutainer blood collection tubes with EDTA anti-coagulant (BD, Franklin Lakes, NJ) *via* venipuncture. Inguinal lymph nodes (LN) were biopsied *via* surgical removal at week 18 following immunization. All the three groups of rhesus macaques were intrarectally (IR) challenged ten times with SHIV-BaL at increasing dose ranging from 1:20 to 1:5.5. Initial challenge strategy designed to infect controls within 3 to 4 exposures. Thus, we performed the challenges with an initial challenge dose with a 1:20 dilution of the virus stock. After 5 challenges, only 4 of 9 control animals were uninfected, and subsequently challenge doses were escalated (for all groups). The remaining 4 controls became infected with concentrations of virus increased to 1:10 and eventually 1:5.5 dilutions.

### ELISA

The binding plasma antibody response to multiple trimeric envelope protein was measured by kinetic enzyme-linked immunosorbent assay (ELISA). For longitudinal ELISA, autologous F8 and heterologous SF162 gp140 proteins were used as the coating antigen and IgG antibody response was measured as previously described (43). For measuring broad plasma binding antibody response, HIV-Envelope gp140 trimeric protein Clade A (KNH1144), Clade B (F8, C6a, PVO4, SF162), Clade C (X1936) Clade D (UG21), Clade F (BR029), HIV-Envelope gp120 trimeric protein Clade AE (A244, 93TH975), Clade B (BaL, MN, JRCSF Fc), Clade C (CN54, 96ZM651), HIV-gp41, RSC3, RSC3G367R, ΔRSC3, RSC3 Δ371I, RSC3 Δ371I/P363N, V1V2 protein Clade AE (A244), and V1V2 protein Clade C (1086) were used. For non-specific antigen ELISA, Pneumovax-23 (MERCK), Tetanus (MassBiologics), Ovalbumin (Ova)(Invivogen), Fluzone (Sanofi) and BSA (Miltenyi Biotec) were used as coating antigen. V3 ladle (V3L) and V3 cradle (V3C) were obtained from Catarina Hioe (44). All other antigens were obtained from the AIDS Reagent Repository unless otherwise mentioned. 96 well flat bottom immunoplates (Thermo Scientific) were coated with the above-mentioned antigens at 0.5 µg/ml overnight at 4°C, blocked with 3% BSA in PBS for 1h, then washed with PBS containing 0.05% Tween 20. Rhesus plasma were diluted to 1:500, 1:2500 and 1:12500 in PBS containing 0.05% Tween 20 and added in duplicates to plates and incubated for 1 h. Plates were washed and plasma antibody binding was detected using anti-rhesus IgG-HRP (NHPRR) at a dilution of 1:2000 and developed by KPL SureBlue TMB substrate. OD values were standardized to PBST OD values that was included with each assay to obtain relative units (RU), and subsequent area under the curve values across the dilutions was determined to calculate the binding plasma antibody response. For determining inhibition of VRC01 binding by plasma, plates were coated with 2 µg/ml of F8 or C6a gp140 recombinant protein overnight, then washed and blocked with 100 µl of Pierce blocking buffer. After 1h at RT, plates were washed and rhesus plasma at a dilution of 1:100 was added to the plates and incubated for 1h at RT. Following wash, biotinylated VRC01 (1 µg/mL) was added and incubated for an hour. After 1 h, plates were washed and bound plasma VRC01 was detected with streptavidin HRP-conjugate (30 min at RT) (Jackson Immunoresearch) and were developed as above.

### TZM-bl Neutralization Assay

The TZM-bl cell neutralization assay was performed as previously described (45). Pseudoviruses were produced using the pSG3ΔEnv DNA plasmid encoding the HIV backbone and a plasmid encoding either homologous or heterologous envelope variants using the jetPEI (Polyplus) transfection reagent. Supernatant containing pseudovirions was harvested after 2–3 days and frozen in 1 ml aliquots at −80°C. Pseudovirus stocks were titrated in TZM-bl cells (NIH AIDS Reagent Program, catalog number 8129) to determine the virus dilution required for 200,000 relative light units (RLU). To measure neutralizing antibody titers, heat-inactivated plasma samples were assayed in duplicate for neutralization against single round of entry Env-pseudoviruses using TZM-bl reporter cells (NIH AIDS Reagent Program, catalog number 8129). Briefly, plasma samples were serially diluted 3-fold in complete DMEM media (DMEM, 10% FBS, L-glutamine) from a starting dilution of 1:50 and incubated with pseudovirus for 1 h at 37°C in 96-well flat bottom plates. TZM-bl cells were harvested, mixed with DEAE dextran (7.5 µg/ml) to enhance viral uptake, and 10,000 cells were added to each well containing antibodies and virus. Additional control wells containing cells and virus only (no antibody) and cells only (no virus, no antibody) were included on each plate. As a positive control, broadly neutralizing monoclonal antibodies were included on every plate in a multi-plate experiment. Plates were incubated at 37°C and 5% CO_2_ for 48–72 h. Bright-Glo (Promega) luciferase substrate was added to each well, and luciferase activity was measured on a luminometer. Wells containing only cells and virus defined 100% RLU signal, and cells-only wells defined 0% RLU signal. To calculate neutralization potency, the RLU in each well was divided by the RLU in the virus-only wells to give the percentage of viral infection not neutralized by antibody. This % RLU was plotted against antibody concentration to generate a dose-response curve for each antibody, from which 50% neutralization titer (IC_50_) could be interpolated.

### Antibody-Dependent Cellular Phagocytosis Assay

ADCP activity of the plasma samples was measured as previously described (46, 47). Pre-immunization and wk34 plasma samples, diluted at 1:50 with PBS for the 1:100 final dilution, were tested for uptake of fluorescent beads coated with HIV Env C6a gp140 by THP-1 cells. Cells were analyzed for fluorescent bead uptake by flow cytometry using a LSRII (BD Biosciences). The phagocytic index of each sample was calculated by multiplying the percentage of bead positive cells (frequency) by the mean fluorescence intensity (MFI) of the beads (degree of phagocytosis) and dividing by 10^6^. Values were normalized with the background values (cells and beads without any plasma) to ensure consistency in values obtained on different assays.

### Antibody-Dependent Complement Deposition Assay

Ab-dependent complement deposition was assessed by measuring complement component C3b on the surface of target cells as previously described in (Richardson et al., 2018) with modifications. Briefly, 250,000 CD4-expressing CEM.NKR target cells (human T-lymphoblastoid cell line obtained from NIH AIDS Reagent Program) were incubated with 1.5 μg of C6a gp140 per reaction for 1 hour at room temperature. Ag-bound cells were washed twice with cold media to get rid of the unbound Ags and incubated with the heat-inactivated test plasma sample for a final dilution of 1:10 in a 5 mL Falcon round bottom tube (Thermo Fisher Scientific, NY, USA) for 30 min at 4°C. An equal volume of HIV-negative serum (Pooled Human Complement Serum, # ICSER10ML, Innovative Research) was diluted with veronal buffer solution containing 0.1% gelatin (Boston Bioproducts Inc., # IBB290) @ 1: 5 for the final 1:10 dilution and added to the tubes. The cells were incubated for 20 min at 37°C and then washed twice with 15 mM EDTA in PBS. For the complement deposition detection, the immune complexes were stained with FITC-conjugated anti-C3/C3b/iC3b antibody (Cedarlane, CL7632F; 1 µl per reaction) and 7-AAD (Invitrogen; A1310; 2 µl per reaction) for 15 min at 4°C and were fixed in 100 μl 4% PFA at the end. The complement deposition was assessed by flow cytometry on the BD LSR II. ADCD score was determined as the percentage of C3b positive cells multiplied by the fluorescence intensity. Values were normalized with the background values (Ag and target cells without any plasma) to ensure consistency in values obtained on different assays and finally were represented as the mean fluorescence intensity (MFI).

### Antibody-Dependent Cellular Cytotoxicity Assay

ADCC activity of plasma collected at week 34 against SHIV SF162P3- and SHIV BaL-infected target cells was determined as previously described (48). In brief, CD4+ CCR5+ NKR24 target cells that express luciferase under control of a tat-dependent promoter were infected with replication-competent SHIV SF162P3 or SHIV BaL (200 ng/ml p27) by spinoculation at 1200 x g for 2 h with 40 mg/ml polybrene. Three days post spinoculation, 1x10^4^ target cells per well were co-incubated with effector KHYG-1 NK cells expressing rhesus CD16 at an effector to target ratio of 10:1, with and without serial plasma dilutions, in 200 µl of assay media (RPMI supplemented with 5 U/ml IL-2) in round-bottom 96-well plates. All plasma dilutions were set up in duplicate. Plates were incubated for 8 h at 37°C and 5% CO_2_, then each assay well was mixed by pipetting. Next, 150 µl was transferred to black flat-bottom plates containing 50 µl of Bright-Glo (Promega) and incubated for 2 min at 25C. Luminescence was measured on a Victor X Light Plate Reader (Perkin Elmer). ADCC activity in samples including serial plasma dilutions was determined as the percentage loss of relative light units compared to that in the no plasma wells using the following formula: [sample mean - background (mock-infected targets and effectors)]/[maximum (SHIV-infected targets and effectors - background)] x 100.

### Immunofluorescence Staining of Lymph Nodes

5 μm paraffin LN sections were incubated at 60°C for 30 minutes and transferred to xylene to remove the paraffin. They were then sequentially hydrated in alcohol, 95% alcohol, and finally immersed in water. Antigens were unmasked by boiling the slides in DAKO antigen retrieval solution (S1699, DAKO) for 30 minutes. Slides with tissue sections were cooled down for 10 minutes and rinsed with distilled water several times. Non-specific binding was blocked with 5% normal donkey serum (017-000-121, Jackson ImmunoResearch Laboratories) in PBS for 30 minutes at room temperature. LN were stained with a combination of primary goat antibodies against PCNA (clone C-20, Santa Cruz Biotechnology), rabbit antibodies specific for human IgD (RB-1436-A1, Thermo Scientific), and mouse antibodies for human CD20 (clone L26, GeneTex) to detect germinal centers. Primary antibodies were visualized with Alexa fluor 568 donkey anti-goat Ig G (A-11057, Thermo Fisher Scientific), FITC donkey anti-rabbit IgG (711-096-152, Jackson ImmunoResearch Laboratories), and Alexa Fluor 647 donkey anti-mouse IgG (715-606-150, Jackson ImmunoResearch Laboratories). Tfh cells were labeled in the LN with goat antibodies specific for human PD1 (AF1086, R&D systems), rat antibodies specific for human CD3 (Clone CD3-12, Bio-Rad) and mouse antibodies against human CD4 (Clone 5D9, MyBioSource). Binding of primary antibodies was revealed by the addition of Alexa fluor 568 donkey anti-goat IgG, Alexa Fluor 488 donkey anti-rat IgG (A-21208, Thermo Fisher Scientific) and Alexa Fluor 647 donkey anti-mouse IgG. Finally, plasma cells (PC) were identified by the typical intracytoplasmic stain and extrafollicular localization with biotin goat anti-monkey IgM (NBP1-73556, Novus Biologicals) and Alexa fluor 488 donkey anti-human IgG (709-546-149, Jackson ImmunoResearch Laboratories), in combination with mouse antibodies against CD20 to label the B cell follicles. Primary antibodies were detected with Alexa Fluor 555 Streptavidin (S32355, Thermo Fisher Scientific) and Alexa Fluor 647 donkey anti-mouse IgG. Immunofluorescently labeled LN sections were washed with PBS and mounted with Vectashield antifade mounting media with DAPI (H-1200, Vector Laboratories). Pictures were taken with a Zeiss Axioplan 2 microscope and recorded with a Hamamatsu camera. All germinal centers were counted in LN paraffin sections from individual animals to estimate the average size of the GC and the number of GC per LN section. Based on the weaker stain with DAPI inside the GC due to the relaxed DNA and taking as a reference the serial sections stained with GC markers, we counted PD1+CD3+CD4+ Tfh outside and inside of GCs, and calculated total numbers of PD1+CD3+CD4+ Tfh in all the GC 200X pictures per animal. Finally, we counted in 5 random 200x fields/LN section, IgG+ and IgM+ PC, and calculated the average number of IgG+ or IgM+ PC per field in each experimental group.

### Flow Cytometry

For total B cell analysis PBMCs were stained for 1 h with antibody cocktail containing anti-human-CD4-BB790 (clone: L200, BD Biosciences), anti-human-CD8-BUV496 (clone: RPA-T8, BD Biosciences), anti-human-CD19-AF700 (clone: J3-119, Beckman Coulter), and anti-human-CD20-APC-Cy7 (clone: L27, BD Biosciences), followed by staining with live/dead blue stain (Molecular Probes). One-to-five million total events per sample were collected on a BD FACS Symphony A5 instrument (BD Biosciences) and analysis were performed with FlowJo software (Treestar, Inc, Ashland, OR). Total PBMC were gated on lymphocytes using FSC and SSC. Live/Dead stain and anti- CD8 and CD4 were used to exclude dead cells, and T cells respectively, and total B cells defined as CD19+CD20+.

To track CD8+ and CD4+ subsets, whole blood was stained with a cocktail containing anti-human-CD95-FITC (clone:DX2, BD Biosciences), anti-human-CD28-PE (clone:CD28.2, Biolegend), anti-human-CD4-APC (clone: GK1.5, Miltenyi Biotec), anti-human-CD8-PB (clone: SK1, Biolegend), anti-human-CD3-AF700 (clone: SP34-2, BD Biosciences), anti-human-CD45-PE-Cy7 (clone: D058-1283, BD Biosciences), and live/dead yellow stain (Molecular Probes). Briefly, whole blood was washed and then stained at room temperature for 30 minutes. Red blood cells were lysed, and the samples were further washed and then fixed with paraformaldehyde. Samples were run on a BD LSRII flow cytometer (BD Biosciences) and analysis performed with FlowJo software (Treestar, Inc, Ashland, OR). Absolute counts of CD4+ and CD8+ T cells and their subset were calculated using lymphocyte/µL from complete blood counts multiplied by the frequencies of CD95+/CD28+ (central memory), CD95+/CD28- (effector memory), and CD95-/CD28+ (naïve).

Quantification of Env-specific Tfh cells was done using an activation induced marker (AIM) assay as described previously (49). In brief, frozen lymph node cells were thawed, washed with AIM-V media (ThermoFisher Scientific) and rested for 3 hours at 37°C. After resting, 2.5 million underwent three conditions: no stimulation, stimulation with 5 µg/mL F8 gp140, or stimulation with 1 µg/mL SEB. After an 18 hour stimulation, cells were stained for 1 hour at 4°C, washed, and fixed with paraformaldehyde, and analyzed as described above. Staining markers were as follows: anti-human-CD25-FITC (clone: BC96, Biolegend), anti-human-PD-1-PerCP-Cy5.5 (clone: EH12.2H7, Biolegend), anti-human-CD45RA-BV421 (clone: 5H9, BD Biosciences), anti-human-OX40-PE (clone: L106, BD Biosciences), anti-human-CD3-AF700 (clone: SP34-2, BD Biosciences), anti-human-CD4-APC (clone: L200, BD Biosciences), anti-human-CD20-APC-H7 (clone: 2H7, BD Biosciences), anti-human-CXCR5-PE-Cy7 (clone: MU5UBEE, ThermoFisher), and live/dead fixable yellow (Molecular Probes). Env-specific Tfh cells were recorded as the fold increase in the frequency of live CD3+ CD4+ AIM+ cells (PD-1+, CXCR5+, OX40+, CD25+) in gp140-stimulated cultures over that in unstimulated cultures.

### Statistical Analysis

Data were analyzed using GraphPad Prism (version 5.0d, GraphPad Software Inc, La Jolla, CA) to calculate statistical significance. A two-tailed unpaired Student’s t-test was used for two-group comparisons. Two-way analysis of variance (ANOVA) with Bonferroni Multiple Comparison test (post-test) was used for comparing multiple groups. Survival analysis of SHIV infected macaques was performed using the logrank test and Wilcoxon test. A p-value <0.05 was considered significant.

## Data Availability Statement

The raw data supporting the conclusions of this article will be made available by the authors, without undue reservation.

## Ethics Statement

The animal study was reviewed and approved by OHSU West Campus Institutional Animal Care and Use Committee.

## Author Contributions

Conceptualization: SS, JR-M, MK, AH, NH, and JK. Performed experiments: SS, PB, SP, DS, WS, MB, RB, JR-M, and JK. Formal analysis: SS, DS, JC, AR, JR-M, AH, and JK. Manuscript preparation: SS, DS, JR-M, AH, and JK. Manuscript review: SS, PB, SP, DS, WS, MB, RB, JC, AR, JR-M, MK, AH, NH, and JK. All authors contributed to the article and approved the submitted version.

## Funding

This research was partially funded by the National Institutes of Health (NIH) (5R01AI117787, 5R01DE027245 to JK; P51-OD011092, U42-OD023038 to NH), and the University of Alabama at Birmingham Center for AIDS Research P30 AI027767.

## Conflict of Interest

The authors declare that the research was conducted in the absence of any commercial or financial relationships that could be construed as a potential conflict of interest.

## Publisher’s Note

All claims expressed in this article are solely those of the authors and do not necessarily represent those of their affiliated organizations, or those of the publisher, the editors and the reviewers. Any product that may be evaluated in this article, or claim that may be made by its manufacturer, is not guaranteed or endorsed by the publisher.
