# Supplementary material for: CD4+ T Cells Are Dispensable for Induction of Broad Heterologous HIV Neutralizing Antibodies in Rhesus Macaques
Source: Front Immunol. 2021 Oct 20;12:757811. doi: 10.3389/fimmu.2021.757811 (PMC8564110; doi:10.3389/fimmu.2021.757811)
Supplement: Supplementary file 1 [file DataSheet_1.pdf]

# *Supplementary Material*

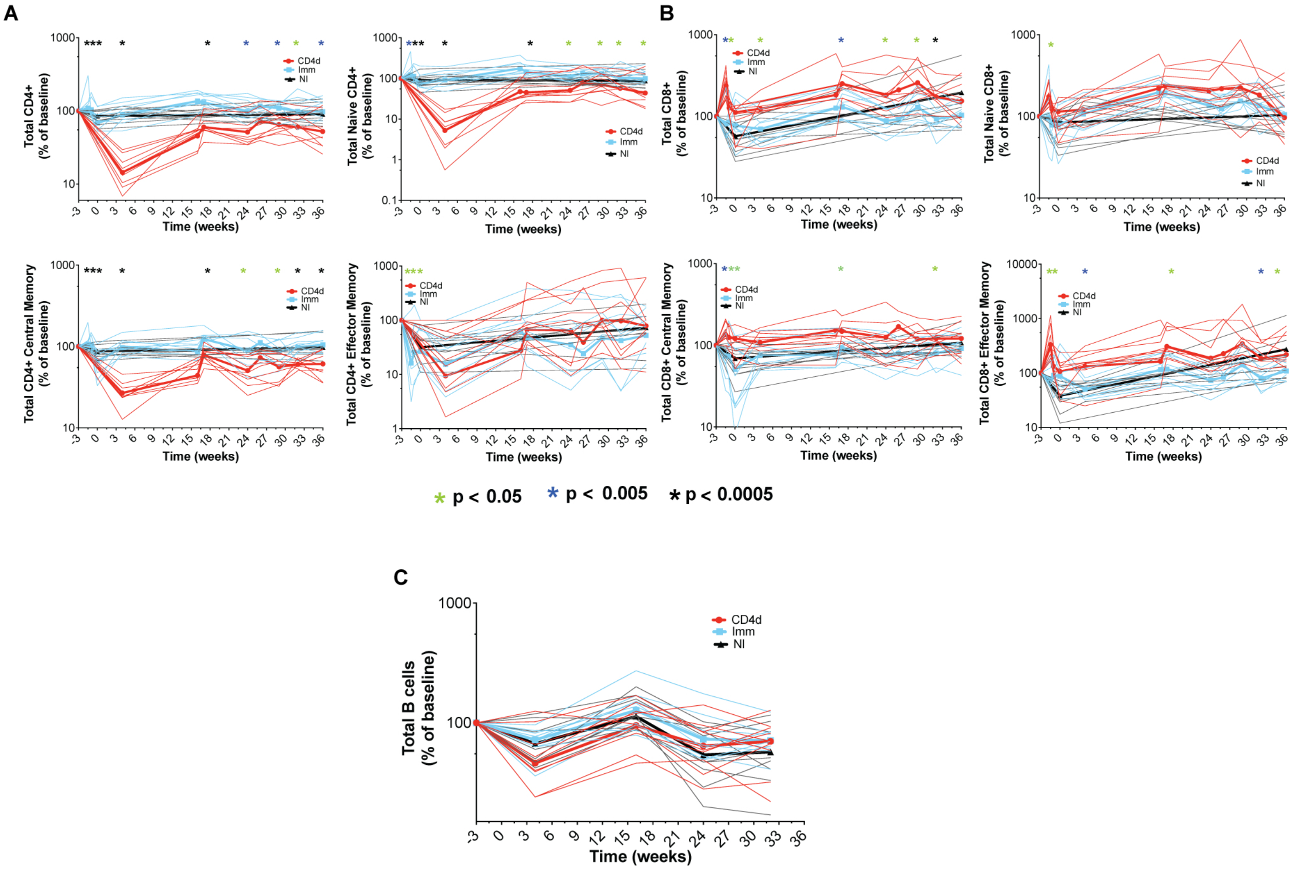


## Supplemental Figure 1

**Frequencies of immune cell subsets in CD4 depleted and vaccinated rhesus macaques.** Flow cytometric analysis of longitudinal distribution of CD4+ **(A)** and CD8+ **(B)** T cells and total CD19+CD20+ B cells **(C)**. Red=CD4 depleted (CD4d) group, turquoise =Immunized (Imm) group, black=Non-Immunized (NI) group. Thin lines represent individual animals, thick lines indicated group mean. Significance determined by the unpaired t-test and are indicated as follows: * $p \leq 0.05$, * $p \leq 0.005$ * $p \leq 0.0005$.

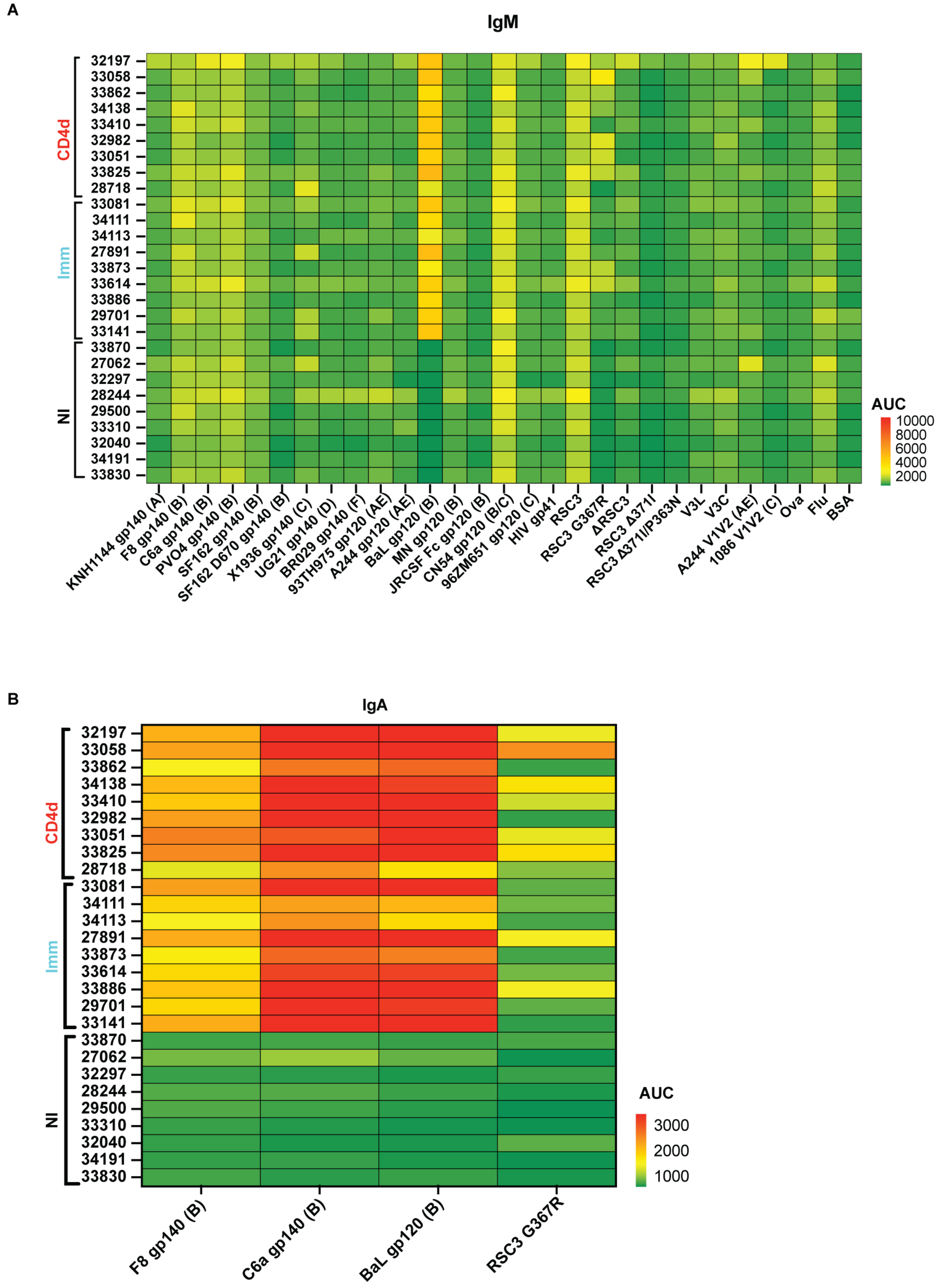


## Supplemental Figure 2

Heat map showing overview of broad HIV-Envelope specific IgM **(A)** and IgA **(B)** response at week 34.

**Supplementary Table 1: Rhesus macaque cohort**

| Group | Rhesus # | Age (years) | Sex | Weight (kg) |
|---|---|---|---|---|
| Immunized+CD4 depletion (CD4d) | 33051 | 4.1 | F | 5.2 |
| | 33058 | 4.0 | F | 5.3 |
| | 33410 | 3.6 | M | 6.1 |
| | 33825 | 3.4 | M | 6.9 |
| | 33862 | 3.5 | F | 4.4 |
| | 28718 | 8.0 | F | 6.6 |
| | 32197 | 4.8 | M | 6.8 |
| | 32982 | 4.6 | M | 8.0 |
| | 34138 | 3.7 | M | 7.0 |
| Immunized Control (Imm) | 27891 | 8.6 | F | 5.5 |
| | 29701 | 7.6 | F | 7.5 |
| | 33081 | 3.7 | F | 5.1 |
| | 33873 | 3.5 | M | 5.9 |
| | 33886 | 3.5 | M | 6.5 |
| | 33141 | 3.9 | M | 6.3 |
| | 33614 | 3.6 | M | 7.4 |
| | 34111 | 3.7 | M | 6.7 |
| | 34113 | 3.7 | M | 6.3 |
| Non-Immunized (NI) | 27062 | 9.7 | F | 6.3 |
| | 28244 | 8.7 | F | 8.3 |
| | 29500 | 7.6 | F | 5.9 |
| | 32040 | 4.7 | M | 8.7 |
| | 33310 | 3.5 | M | 6.4 |
| | 32297 | 4.6 | M | 6.5 |
| | 33830 | 0.4 | M | 6.1 |
| | 33870 | 3.6 | M | 6.6 |
| | 34191 | 3.3 | M | 5.4 |
